# Identification and Expression Analysis of TCP Transcription Factors Under Abiotic Stress in *Phoebe bournei*

**DOI:** 10.3390/plants13213095

**Published:** 2024-11-03

**Authors:** Wenzhuo Lv, Hao Yang, Qiumian Zheng, Wenhai Liao, Li Chen, Yiran Lian, Qinmin Lin, Shuhao Huo, Obaid Ur Rehman, Wei Liu, Kehui Zheng, Yanzi Zhang, Shijiang Cao

**Affiliations:** 1 College of Jun Cao Science and Ecology (College of Carbon Neutrality), Fujian Agriculture and Forestry University, Fuzhou 350002, China; lwz77557575@163.com; 2 College of Forestry, Fujian Agriculture and Forestry University, Fuzhou 350002, China; m15803366858@163.com (H.Y.); qiumianzheng@163.com (Q.Z.); lwh1623850793@163.com (W.L.); chenli4210@126.com (L.C.); 3 College of Life Sciences, Fujian Agriculture and Forestry University, Fuzhou 350002, China; 18358678977@163.com (Y.L.); 18760030926@163.com (Q.L.); 4 School of Food and Biological Engineering, Jiangsu University, Zhenjiang 212013, China; huo@ujs.edu.cn (S.H.); obaid.sheikh@hotmail.com (O.U.R.); 5 Shandong Analysis and Test Center, Qilu University of Technology (Shandong Academy of Sciences), Jinan 250014, China; liuweics@qlu.edu.cn; 6 College of Computer and Information Sciences, Fujian Agriculture and Forestry University, Fuzhou 350002, China; zhkehui@fafu.edu.cn; 7 Metabolomics Center, Haixia Institute of Science and Technology, Fujian Agriculture and Forestry University, Fuzhou 350002, China

**Keywords:** TCP gene, *Phoebe bournei*, identification, abiotic stress, expression analysis

## Abstract

The *TCP* gene family encodes plant transcription factors crucial for regulating growth and development. While *TCP* genes have been identified in various species, they have not been studied in *Phoebe bournei* (Hemsl.). This study identified 29 *TCP* genes in the *P. bournei* genome, categorizing them into Class I (PCF) and Class II (CYC/TB1 and CIN). We conducted analyses on the *PbTCP* gene at both the protein level (physicochemical properties) and the gene sequence level (subcellular localization, chromosomal distribution, phylogenetic relationships, conserved motifs, and gene structure). Most *P. bournei TCP* genes are localized in the nucleus, except *PbTCP9* in the mitochondria and *PbTCP8* in both the chloroplast and nucleus. Chromosomal mapping showed 29 *TCP* genes unevenly distributed across 10 chromosomes, except chromosome 8 and 9. We also analyzed the promoter cis-regulatory elements, which are mainly involved in plant growth and development and hormone responses. Notably, most *PbTCP* transcription factors respond highly to light. Further analysis revealed three subfamily genes expressed in five *P. bournei* tissues: leaves, root bark, root xylem, stem xylem, and stem bark, with predominant *PCF* genes. Using qRT-PCR, we examined six representative genes—*PbTCP16*, *PbTCP23*, *PbTCP7*, *PbTCP29*, *PbTCP14*, and *PbTCP15*—under stress conditions such as high temperature, drought, light exposure, and dark. *PbTCP14* and *PbTCP15* showed significantly higher expression under heat, drought, light and dark stress. We hypothesize that *TCP* transcription factors play a key role in growth under varying light conditions, possibly mediated by auxin hormones. This work provides insights into the *TCP* gene family’s functional characteristics and stress resistance regulation in *P. bournei*.

## 1. Introduction

Plants, being sessile organisms, are exposed to various degrees of abiotic stresses during their growth and development. These stresses arise when the external environmental conditions become unsuitable and deviate from the optimal range required for plant growth. Consequently, plants transduce environmental signals into internal signals to regulate physiological processes through numerous regulatory proteins, enabling continual adaptation to changing conditions [1,2]. Transcription factors (TFs), as essential proteins, play a crucial role in plant stress responses by interacting with *cis*-elements to regulate gene expression within specific DNA sequences of target gene promoters [3,4].

The *TCP* gene family, first identified in 1990, derives its name from the initials of three members: *Teosinte Branched1* (*TB1*) in maize (*Zea mays*), *Cycloidea* (*CYC*) in snapdragon (*Antirrhinum majus*), and *Proliferating Cell Factors 1* and *2* (*PCF1 and PCF2*) in rice (*Oryza sativa*) [5,6,7,8]. The *TCP* domain comprises 59 amino acids and features an atypical basic helix–loop–helix (bHLH) motif at the N-terminus, crucial for mediating protein–protein interactions through the coiled helix structure [9]. The protein crystal structure of rice *TCP OsPCF6* reveals that the *TCP* domain forms a dimer, maintaining a stable conformation [10].

Based on differences within their domains, *TCPs* are categorized into two main classes: Class I (PCF subfamily) and Class II (CIN and CYC/TB1 clades). Class I domains lack four amino acids, whereas the Class II CYC/TB1 clade features an arginine-rich R domain [11,12,13]. Research indicates that these clades exhibit distinct DNA recognition mechanisms. Class I *TCPs* bind to GGNCCC or CCNNCC sequences, while Class II *TCPs* specifically bind to the GGNCCC sequence [14,15]. Functionally, Class I transcription factors are involved in leaf and flower organ development, seed germination, and cell proliferation, thereby promoting plant growth. Conversely, Class II transcription factors are associated with hormone regulation, defense responses, and branching regulation, inhibiting plant growth [16,17,18].

**Figure 1:**
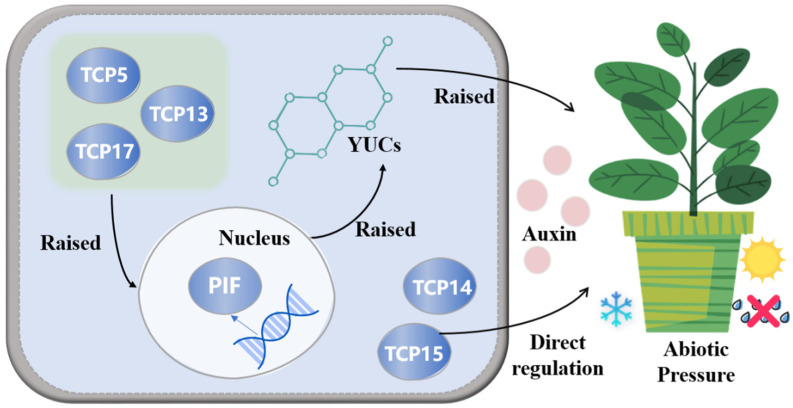
Conceptual framework of regulation of auxin signaling by *TCP* transcription factors. Note: The role of *TCP* transcription factors in regulating auxin biosynthesis and plant responses to abiotic stress. *TCP5*, *TCP13*, and *TCP17* enhance auxin synthesis by upregulating PIF, which in turn increases the expression of YUC enzymes, key players in the auxin biosynthesis pathway. Elevated auxin levels contribute to plant growth and stress adaptation. *TCP14* and *TCP15* specifically promote plant elongation by regulating auxin-induced genes associated with cell expansion. Together, these *TCP* factors support plant resilience under various abiotic stresses.

*TCP* transcription factors play significant roles in seed germination, leaf senescence, defense responses, circadian rhythm regulation, and cell proliferation by modulating plant hormones [9,19,20,21,22]. Auxin, a vital hormone, influences organ size, differentiation, and morphology, serving as a key player in plant growth, development, and stress responses [23]. YUC enzymes, which are rate-limiting enzymes in the tryptophan-dependent auxin biosynthesis pathway, are regulated by the PIF transcription factor, which promotes shade response gene expression under low-light conditions. *TCP17*, *TCP5*, and *TCP13* enhance auxin signaling by upregulating PIF and YUCs, thereby boosting auxin biosynthesis [24]. Additionally, *TCP14* and *TCP15* are known to promote proper plant elongation by regulating a set of auxin-induced genes associated with cell expansion (Figure 1) [25]. Therefore, *TCP* transcription factors are crucial in the regulation of auxin biosynthesis.

The *TCP* gene family has been identified in various plants [26], including *Arabidopsis thaliana* with 24 *TCP* transcription factors [22], tomato (*Solanum lycopersicum*) with 30 *TCP* genes [27], *Dendrobium streptolum* with 25 transcription factors [11], and oats (*Avina sativa*) with 49 *TCP* genes [28]. In ginseng (*Panax ginseng*), the *PgTCP26-02* gene is involved in ginsenoside synthesis and regulates secondary metabolism [29]. The *CaTCP16* gene mediates bud phylogeny in pepper (*Capsicum annuum*) [3], and NGAs regulate auxin-related genes in apical pistils through their interaction with *TCP4* [30]. In tomato, *SlTCP12*, *SlTCP15*, and *SlTCP18* influence fruit ripening [1], while *PavTCP1*, *PavTCP2*, and *PavTCP3* affect total anthocyanins, soluble sugars, and soluble solids in sweet cherries (*Prunus avium*) [31]. Leaf development in larch (*Larix decidua*) is regulated by the interaction of class I and class II *LcTCP* genes [32]. In birch (*Betula pendula*), *BpTCP20* enhances salt tolerance by regulating stomatal closure and reducing reactive oxygen species (ROS) accumulation [33]. Additionally, *PeTCP* expression in passionflower is induced by hormone treatment and various stresses such as cold, heat, and salt [34]. The diverse biological functions of *TCP* genes warrant further study due to their extensive roles.

Forests are the key to the global carbon balance and are a key solution to climate change [35]. Compared to plantations, natural forests have more sustained and stronger carbon sequestration capabilities [36]. *Phoebe bournei* (Hemsl.), one of the most valuable native tree species in southern China, is widely used in shipbuilding, woodworking, and other industries due to its excellent properties, providing significant ecological and economic benefits [37,38]. However, overexploitation and its inherently slow growth have led to a decline in *P. bournei* habitat adaptability [39], and global warming is exacerbating abiotic stresses such as high temperatures and drought, which adversely affect its physiological activities [40]. Studies have shown that different light stress conditions impact the leaf area, chlorophyll a, and chlorophyll b in *P. bournei* [41,42]. Increases in temperature and light intensity can enhance the emission of biogenic volatile organic compounds (BVOCs) from *P. bournei*, prolonging the atmospheric residence time of greenhouse gases like CH_4_ and creating a positive feedback loop [43]. Drought stress inhibits the synthesis of Chla and Chlb in *P. bournei*, reducing photosynthesis, and under prolonged drought conditions, stem weight decreases significantly, and seedlings undergo extensive transcriptomic changes [44]. Therefore, understanding the mechanisms underlying *P. bournei* response to abiotic stress is crucial.

Despite extensive research demonstrating that *TCP* transcription factors play a key role in plant growth, development, and stress response, no systematic analysis has been conducted in *P. bournei*. In this study, we used bioinformatics and expression analysis to investigate TCP transcription factors in *P. bournei*. We analyzed the physicochemical properties, gene structure, chromosomal distribution, exon–intron organization, conserved motifs, and promoter regions of the TCP gene family. Additionally, we employed qRT-PCR to assess their expression patterns under high temperature, drought, and light stress conditions. The findings will contribute to understanding the functional characteristics and potential roles of the *TCP* gene family, providing high-quality candidate genes for genetic engineering in *P. bournei* at the molecular level.

## 2. Results

### 2.1. Identification of the PbTCP Protein Characterization

**Figure 2:**
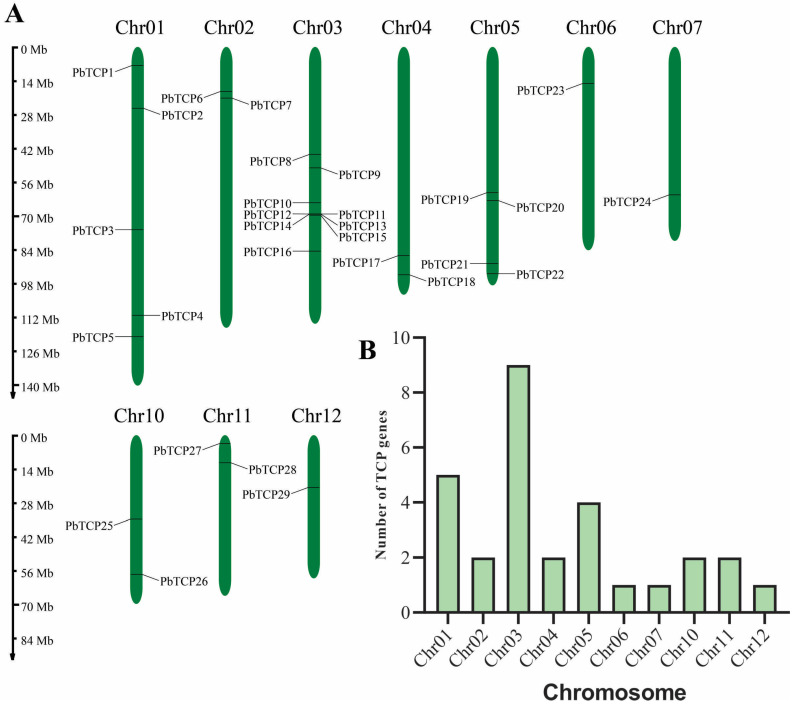
Chromosomal localization analysis of the *TCP* gene family of *Phoebe bournei* (Hemsl.). Distribution of *PbTCP* genes in the *P. bournei* chromosome. (**A**) Each chromosome figure shows the chromosome number at the top. The scale on the left can be used to assess chromosome length and gene position. (**B**) The number of *TCP* genes on the chromosome.

We identified 29 *PbTCP* proteins from the *P. bournei* genome database, naming them *PbTCP1* to *PbTCP29* (Table 1). Analysis revealed that the amino acid (aa) lengths of the *TCP* gene family members ranged between 177 and 679 amino acids (aa) and had an average length of 362 aa. The relative molecular weights of these proteins varied between 17.80 kDa (*PbTCP10*) and 72.15 kDa (*PbTCP9*), averaging 39.15 kDa, indicating significant variability in the size of *TCP* transcription factor proteins. The isoelectric points of these proteins range from 5.68 to 10.15, with nine of the 29 *TCP* proteins being slightly acidic, while the remaining 20 are basic. Except for *PbTCP9*, all identified proteins are considered unstable (instability index > 40), with a range from 37.11 to 69.1. The aliphatic index of *TCP* proteins ranges from 51.61 to 92.99, with an average value of 68.38, suggesting that these proteins are thermostable. Additionally, all *TCP* proteins are hydrophilic (GRAVY < 0), indicating strong hydrophilicity. Subcellular localization predictions show that most *PbTCP* family members are in the nucleus (Table 1), except *PbTCP9*, which is found in the mitochondria, and *PbTCP8*, which is located in both the chloroplast and the nucleus. This suggests that the primary regulatory function of these proteins occurs in the nucleus. Chromosomal localization indicates that the 29 *TCP* genes are unevenly distributed across 10 chromosomes, with no *TCP* genes found on chromosomes 8 and 9 (Figure 2). Notably, chromosome 3 contains the highest number of genes, with nine (*PbTCP8* to *PbTCP16*), which are closely arranged in this region, occupying approximately 43 Mb. Chromosomes 6, 7, and 12 each contain a single gene, with some located near the chromosome ends.

### 2.2. Evolutionary Analysis of PbTCP Gene Family

**Figure 3:**
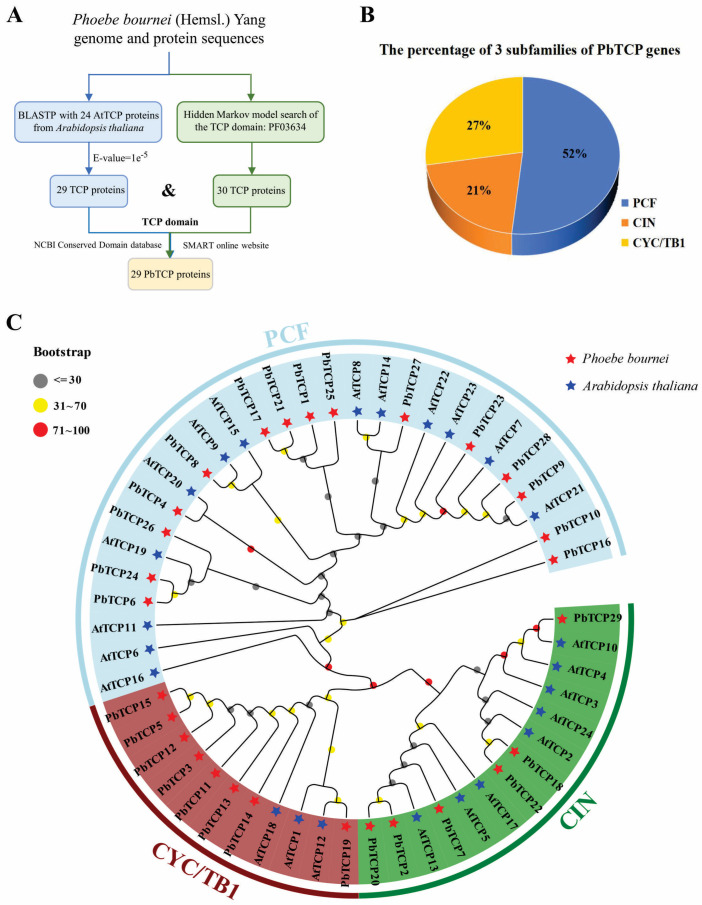
Phylogenetic analysis of *TCP* protein. (**A**) Genome and protein sequences *P. bournei*. (**B**) The percentage of 3 subfamilies of PbTCP genes. (**C**) Phylogenetic tree of *PbTCP* and *AtTCP* proteins. The arcs of different colors indicate a subfamily of the TCP family. One thousand times with MEGA11 and Bootstrap respectively. The tree was constructed by 29 *PbTCPs* identified in *P. bournei* and 25 *AtTCPs* identified in *A. thaliana*.

To investigate the evolution of the *PbTCP* gene family, we constructed a phylogenetic tree using protein sequences from *P. bournei* and *A. thaliana* (Figure 2). Based on the multiple sequence alignment of 29 *PbTCP* and 24 *AtTCP* proteins, all *TCP* proteins were classified into three branches: PCF, CIN, and CYC/TB1, belonging to the two subfamilies, Class I *TCP* and Class II *TCP*. Among these, the PCF branch contains 15 *PbTCP* proteins, representing 52% of the total *PbTCP* proteins, making it the largest among the four subfamilies. The CIN subfamily is the smallest, with only six proteins. Although the CYC/TB1 subfamily has a modest number of proteins (eight), *PbTCP* proteins constitute a significant proportion (73%). In comparison, the PCF subfamily in *A. thaliana* has 13 *AtTCP* genes, the CIN subfamily has eight, and the CYC/TB1 subfamily has three, indicating that the number of *TCP* genes in each subfamily varies between species (Figure 3). The study also reveals that the evolution rate of *PbTCP* genes is higher than that of *AtTCP* genes, resulting in a greater diversity of gene types. This accelerated evolution in *P. bournei* may be an adaptive response to environmental changes, enhancing the species’ survival rate.

### 2.3. PbTCP Protein Sequence Analysis

**Figure 4:**
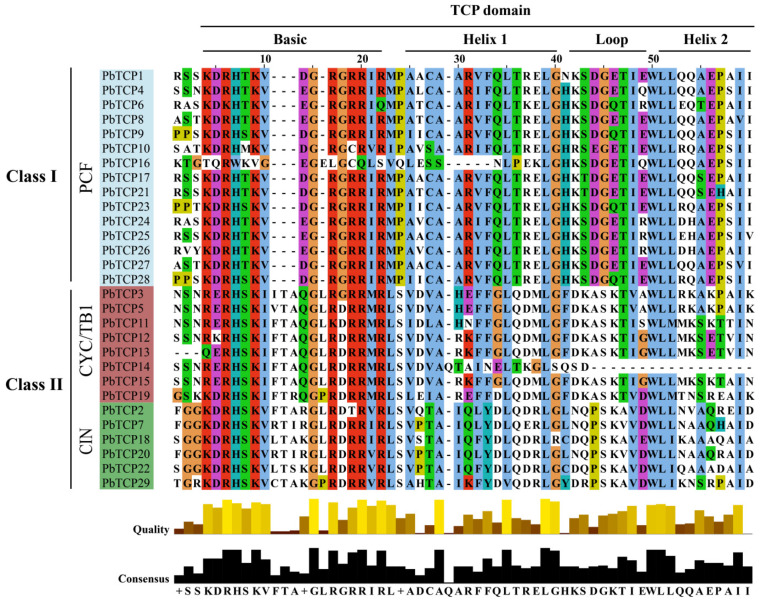
Multiple sequence alignment of TCP domains. Note: TCP domain serial alignment of *P. bournei* TCP family members. At the bottom, the highly conserved amino acid position is indicated by the length of the rectangle; The serial indicator is displayed at the bottom.

We further analyzed the sequence characteristics of the 29 *PbTCP* proteins by comparing their conserved domain sequences (Figure 4). The results reveal that *PbTCP* proteins contain four conserved motifs: basic, helix I, loop, and helix II. The degree of stacking suggests that these motifs are highly conserved within the same subfamily. Compared to Class II, Class I is missing four amino acids in the basic region but exhibits higher overall conservation, allowing for effective differentiation between subfamilies. The basic region is the most conserved, followed by the helix regions, while the loop region shows the greatest diversity. Specific amino acids, such as glycine (G) in the basic region, leucine (L) in the helix region, and tryptophan (W), are completely conserved across all sequences. These residues likely play critical roles in protein function by participating in different structural domains. Additionally, the *PbTCP14* protein exhibits significant amino acid deletions in both the loop and helix II regions, which may contribute to functional differences between Class I and Class II proteins, potentially affecting their regulatory roles and the expression of target genes.

### 2.4. Protein Structure Analysis of PbTCP Transcription Factors

**Figure 5:**
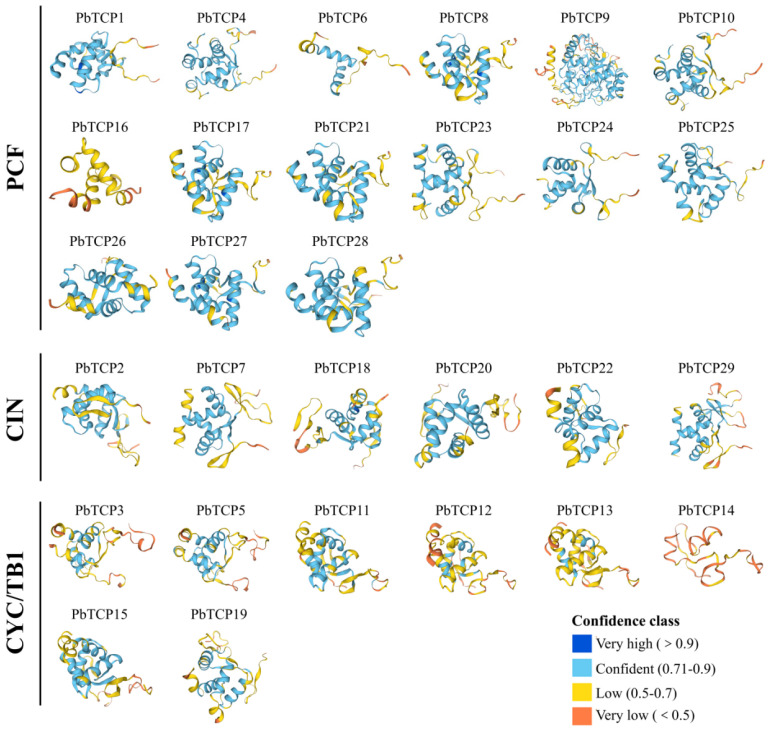
*PbTCP* protein structure analysis. Note: The categories of three branches are marked on the left, and the confidence level of the protein’s secondary structure is indicated by different colors, and the four levels of confidence are shown in the lower right corner.

**Figure 6:**
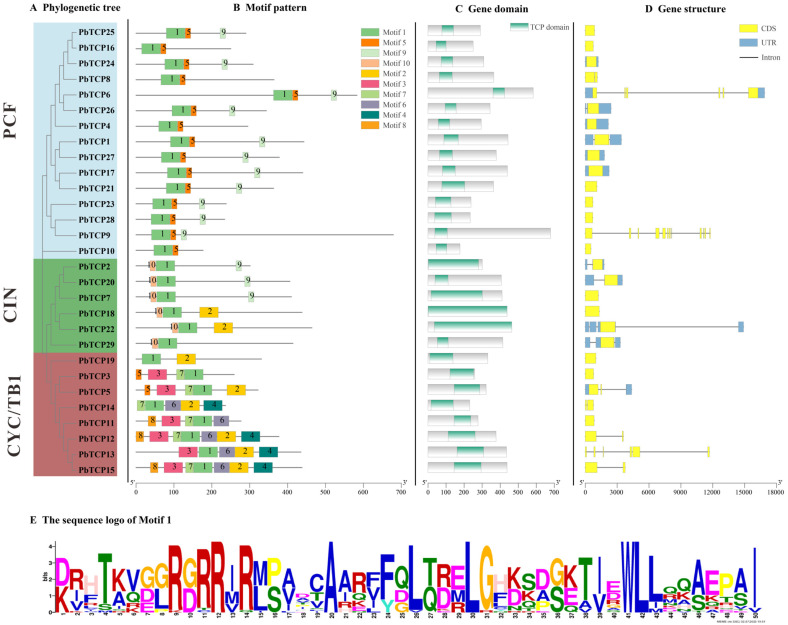
*PbTCP* conserved domain and motif analysis. (**A**) Phylogenetic tree of *PbTCPS*. (**B**) The motif of *PbTCPS*. Patterns 1–10 are displayed in rectangles of different colors. Protein length can be estimated using the scale at the bottom. (**C**) *PbTCP* protein with conserved domains. (**D**) Gene structure of the *PbTCPS* gene. Yellow boxes indicate exons (CDS), black lines indicate introns, and blue boxes indicate 5′ and 3′ untranslated regions. (**E**) The sequence logo of Motif1.The colored letters indicate the specific sequence of motif1.

We conducted a secondary structure analysis of all *PbTCP* proteins, using color coding to represent different levels of confidence. As shown in Figure 5, the PCF branch exhibited a higher confidence level compared to CIN and CYC/TB1 branches. The distinct structures of different proteins further confirm the diverse functional expression of the *TCP* gene family. The tertiary structure was further predicted, and a total of 10 conserved motifs were identified, which were named motif1–motif10. Comparative analysis revealed that these motifs were conserved across different subfamilies. All *PbTCP* proteins contain motif 1, indicating that they were highly conserved. The members from the PCF subfamily all contained motif5, and the motifs within the family were highly similar. The members of the CIN subfamily consistently contain motif 10 (Figure 6), which is located at the N-terminus, while motif 9 was only present in the PCF and CIN subfamilies and located in the C-terminus (Figure 6). The special location may be related to a specific function, but the specific role remains unclear.

In addition, motifs 3 and 7 were only found in the CYC/TB1 subfamily, specifically in *PbTCP12*, *PbTCP13*, *PbTCP14* and *PbTCP15*, and motifs 4 and 6 were found in the CYC/TB1 subfamily, indicating the diversity and uniqueness of the CYC/TB1 subfamily (Figure 6). In the comparison of the exon and intron structure of the *PbTCP* gene family, it was found that all 29 genes contained exons of different lengths, and 52% of *PbTCP* did not have introns.

### 2.5. Collinearity Analysis of TCP Gene Family in P. bournei

**Figure 7:**
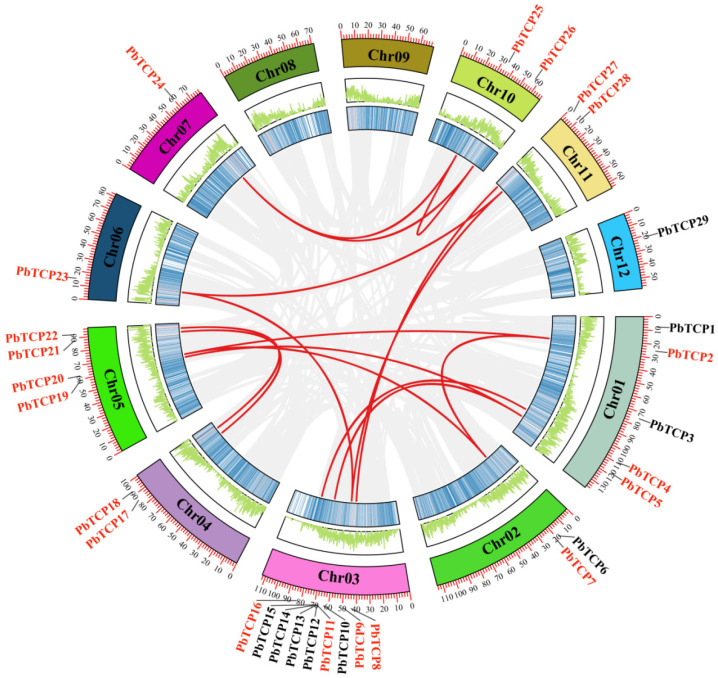
Genomic location, replication events, and homology of the *PbTCP* gene. Note: Synteny analysis of the *PbTCP* family in *P. bournei*. The gray line represents all isotope blocks in the *P. bournei* genome, while the red line represents the gene pairs of the duplicate *PbTCP*. The chromosome number is displayed in a rectangular box for each chromosome.

Intraspecific collinearity analysis of the *PbTCP* gene was performed. Twenty-nine *PbTCP* genes were mapped to 10 chromosomes except chromosomes 8 and 9 (Figure 7). A total of 15 pairs of fragments of repeat events were found, involving 20 *PbTCP* genes. The *TCP* gene on chromosome 3 had the most genes (9) and was involved in most of the repeat events (five pairs, among which *PbTCP9* involves two pairs with *PbTCP23* and *PbTCP28* respectively). Most of the remaining duplication events occurred on chromosomes 1 and 5. Among them, nine pairs of collinearity appeared in the PCF genome, four pairs appeared in the CIN genome, and only two pairs appeared in the CYC/TB1 genome. It is worth noting that many *PbTCP* genes have a collinearity relationship with more than one pair of genes, suggesting that *TCP* genes are highly conserved during evolution in *P. bournei*. In addition, no tandem duplication events were analyzed. The results of collinearity analysis revealed the functional diversity of *TCP* transcription factors in *P. bournei*.

### 2.6. Interspecific Collinearity Analysis

**Figure 8:**
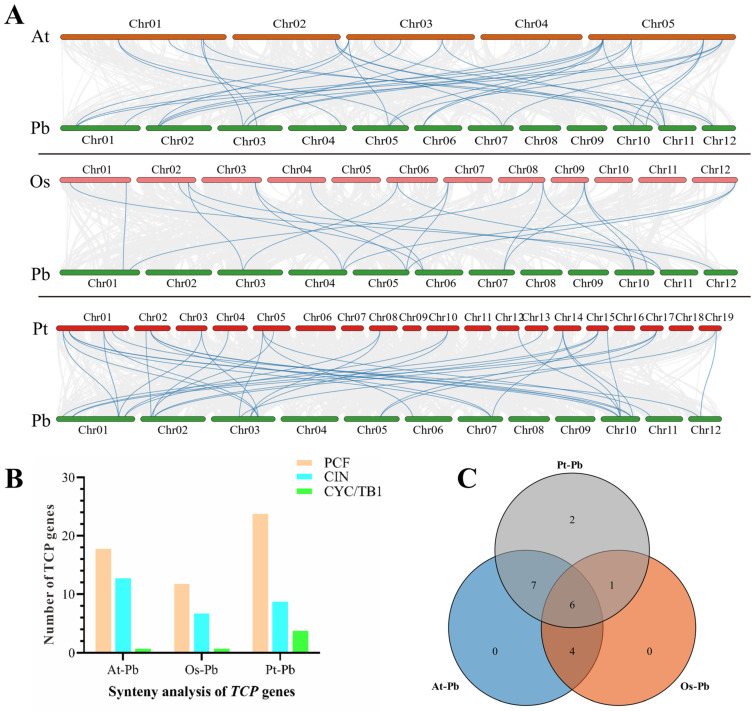
Orthologous analysis of TCP genes in *A. thaliana*, *O. sativa*, *P. trichocarpa* and *P. bournei.* (**A**) Genome homology analysis of *A. thaliana* and *P. trichocarpus*. The grey line represents the genome pairs between homologous blocks, and the blue line highlights the *TCPS* gene pairs synthesized in the three species. (**B**) Number of genome pairs of three clades of different species. (**C**) Number of shared genes of three genome pairs of three species.

To further explore the evolutionary mechanisms, an inter-species collinearity analysis of *TCP* gene pairs was conducted between the genomes of *P. bournei* and *A. thaliana*, *O. sativa*, and *Populus trichocarpa* (black cottonwood) (Figure 8A). The results indicate that the strength of association with *PbTCP* genes, from highest to lowest, is as follows: *AtTCP*, *PtTCP*, and *OsTCP*. Most collinear relationships are found within the PCF subfamily, followed by the CIN subfamily. Consistent with previous analyses, the CYC/TB1 branch shows lower similarity across species. Furthermore, 17 *PbTCP* genes were found to be homologous with those in *A. thaliana*, 11 with *O. sativa*, and 16 with *P. trichocarpa*. Six genes are shared among all three species. Notably, two unique homologous genes (Figure 8) were absent in *A. thaliana* and *O. sativa*, which may be related to the evolutionary history of plant genomes (Figure 8), as well as factors such as gene family expansion and recombination.

### 2.7. Analysis of Cis-Acting Elements

**Figure 9:**
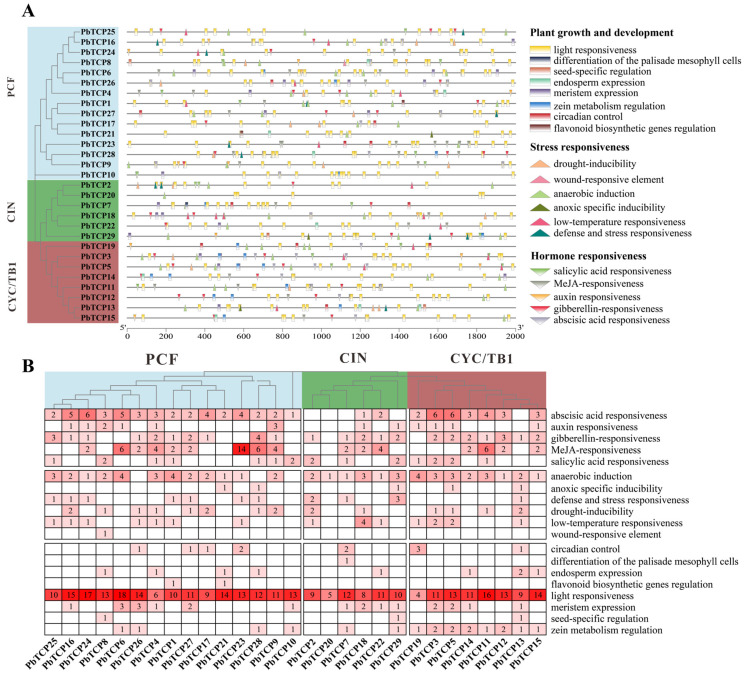
Analysis of the cis-acting element of the gene for the promoter. (**A**) Cis-component predictions of 29 *PbTCP* gene promoter serial (−2000 bp) were analyzed using PlantCARE technology. Here are the 19 categories of cis-elements. (**B**) Number of 19 cis-components for the 29 *PbTCP* genes.

To clarify the role of cis-acting elements in the *PbTCP* promoter regions in light response and environmental stress response processes, we identified and aligned the cis-acting elements present in the *PbTCP* promoters. A total of 723 cis-acting components belonging to 19 functional groups/types were identified in the *TCP* gene promoter serial (Figure 9). There were eight types of growth and development response elements, six stress response elements and five hormone response elements. Among them, the number of plant growth and development elements was the largest, with a total of 398, accounting for 55%; followed by hormone-responsive elements, accounting for 29%, while stress-responsive elements accounted for only 16% (Figure 9A). It is worth noting that cis-elements related to light response function were widely distributed in *PbTCP* gene promoters, indicating that *TCP* genes play an important role in the light response process of plants. Also, elements related to hypoxia-specific induction, circadian rhythm, and differentiation of palisade mesenchymal cells were abundant in 29 *PbTCP*. In addition, abscisic acid response elements and methyl jasmonic acid elements were also widely distributed in the *TCP* genes of *P. bournei*, suggesting that the *TCP* genes may be involved in the regulation of the signaling processes of these two plant hormones, plant stress, and plant growth and development (Figure 9B).

### 2.8. Gene Expression Heat Map of TCP Gene in Different Tissues

**Figure 10:**
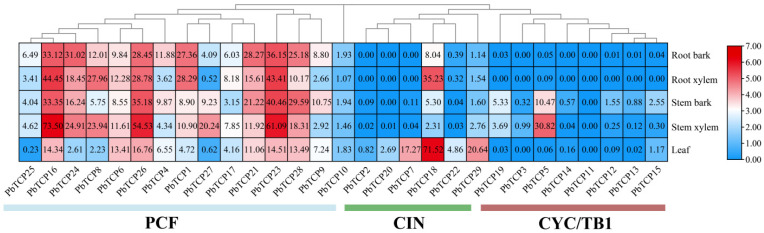
Expression spectrum of *PbTCP*. Note: Different colors are used to indicate the level of expression, and there is an expression value on the right. At the bottom, there are three sub-categories with gene names.

Transcriptome data analysis from five different tissues—leaves, root bark, root xylem, stem xylem, and stem bark—reveals significant tissue-specific expression of *PbTCP* genes in *P. bournei*. The results show that PCF genes are expressed at much higher levels than CYC/TB1 genes. Among the PCF genes (Figure 10), *PbTCP16*, *PbTCP26*, and *PbTCP23* exhibit high expression levels in both roots and stems, indicating strong tissue specificity and suggesting that these three *TCP* genes may play crucial roles in the growth and development of *P. bournei*. In contrast, CYC/TB1 genes generally show low expression across roots, stems, and leaves. Within the CIN genes, *PbTCP2*, *PbTCP20*, and *PbTCP7* have zero expression in roots and very low expression in stems. Notably, *PbTCP18* is highly expressed in leaves and root xylem, suggesting its importance in these tissues. Among the CYC/TB1 genes, *PbTCP5* also exhibits higher expression in the stem xylem, which may be closely related to the growth of the stem xylem. The remaining *TCP* genes have very low expression levels across roots, stems, and leaves (Figure 10).

### 2.9. Expression of the PbTCP Gene Under Abiotic Stress

**Figure 11:**
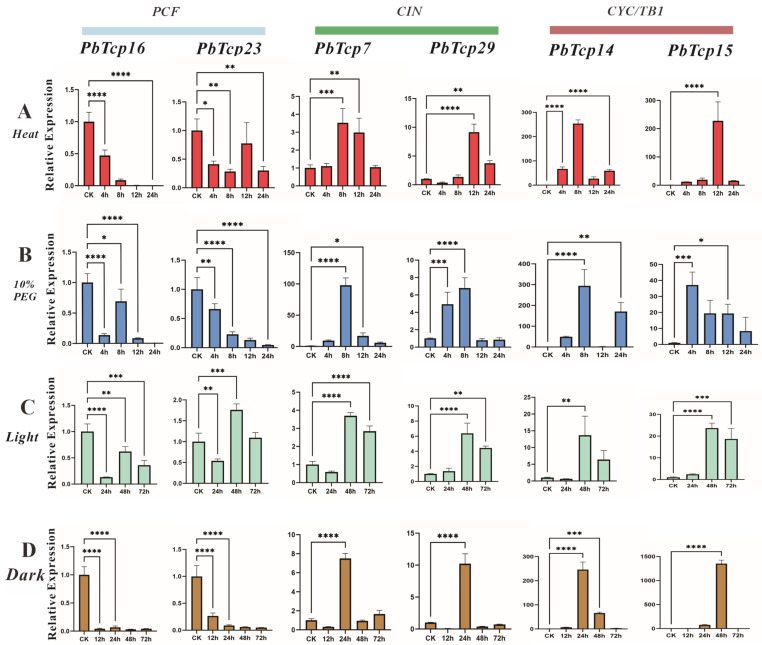
The expression of *PbTCPs* under high temperature, drought, light stress and dark stress was detected by qRT-PCR. (**A**) Relative gene expression levels at high temperature (40 °C) and control (25 °C). (**B**) Relative gene expression levels at the same point (4, 8, 12, and 24 h) were treated with 10% PEG nutrient solution in a simulated arid environment. The control group is treated in distilled water. (**C**) Relative gene expression levels under light stress. (**D**) Relative gene expression levels under dark stress. (* *p* < 0.05, ** *p* < 0.01, *** *p* < 0.0005, **** *p* < 0.0001).

Six genes were selected from each of the three subfamilies for analysis under four stress conditions: high temperature, drought, light stress, and shading (Figure 11). *PbTCP23* and *PbTCP16* are members of the PCF subfamily; *PbTCP7* and *PbTCP29* belong to the CIN subfamily; and *PbTCP15* and *PbTCP14* are part of the CYC/TB1 subfamily. The results indicated that all stress conditions significantly impacted *PbTCP* gene expression, with distinct responses observed across the PCF, CIN, and CYC/TB1 subfamilies.

In the PCF subfamily, *PbTCP14* showed a marked increase under drought stress (simulated by immersion in a 10% PEG6000 solution to mimic short-term drought conditions), with expression levels rising approximately 300-fold at 8 h post-treatment compared to controls. Similarly, *PbTCP7* displayed an approximately 100-fold increase at this point, indicating a robust upregulation response to drought stress in this subfamily. Further studies extending treatment duration and measuring relative water content could provide deeper insights into the drought response of these woody plants. In the CIN subfamily, *PbTCP23* and *PbTCP16* showed varying expression suppression under shading stress, suggesting that CIN members may be particularly sensitive to light reduction. For *PbTCP7, PbTCP29*, *PbTCP14*, and *PbTCP15*, upregulation was observed approximately 24 h after exposure to high temperature and light stress, with CYC/TB1 subfamily genes (*PbTCP14* and *PbTCP15*) exhibiting a delayed yet strong response. This pattern suggests that the CYC/TB1 subfamily may be especially responsive to prolonged stress exposure. Overall, these findings indicate that PCF subfamily genes typically respond rapidly to drought, CIN subfamily genes are more sensitive to shading, and CYC/TB1 subfamily genes exhibit delayed but substantial expression changes under high temperature and light stress.

## 3. Discussion

Plants encounter various abiotic stresses during growth, sparking interest in how they regulate their signaling pathways under such conditions. *TCP*, a plant-specific transcription factor gene family, plays a crucial role in development, influencing traits such as floral symmetry, leaf morphology, plant branching, and hormone signaling [26]. While *TCP* genes have been extensively studied in various plants, including *A. thaliana* [22], *Chrysanthemum morifolium* [13], *Dendrobium officinale* [45], *Catharanthus roseus* [46], and *Citrus sinensis* [47], they have not yet been identified and characterized in *P. bournei*.

In this study, we identified 29 *TCP* genes from *P. bournei*, classified into two subfamilies: Class I (PCF) and Class II (CIN and CYC/TB1), consistent with findings in other species like *Helianthus annuus* [12], *Zingiber officinale* [48], *Dendrobium officinale* [49], *Petunia hybrida* [49], and *Melastoma candidum* [50]. This conservation suggests that *TCP* genes in *P. bournei* are likely to perform similar functions to those in other species. Subcellular localization analysis revealed that all *P. bournei TCP* family members, except *PbTCP8* and *PbTCP9*, primarily function in the nucleus. *PbTCP8* is expressed in both chloroplasts and the nucleus, like *SsTCP13* in sugarcane and *SbTCP15* in sorghum [51,52]. Notably, *PbTCP8* shows high expression in root epidermis, root xylem, stem epidermis, and stem xylem, suggesting a potential role in chloroplast regulation. *PbTCP9*, the only stable protein among the *TCP* genes, is predominantly expressed in mitochondria and demonstrates the highest heat stability, indicating its ability to maintain function under abiotic stress and support mitochondrial stability. However, the regulatory mechanism of *PbTCP9* requires further investigation.

Structural similarities across *TCP* genes are evolutionarily conserved and functionally significant. The presence of conserved motifs and gene structures supports this conclusion. For instance, motif 1 is widespread among *PbTCP* genes, while *PbTCP1* (PCF), *PbTCP2* (CIN), and *PbTCP3* (CYC/TB1) share similar motifs. *PbTCP18* (CIN) and *PbTCP19* (CYC/TB1) have similar exon lengths, indicating that genes from different branches may have similar functions, preventing phenotypic changes due to single gene mutations under abiotic stress. Additionally, each subfamily possesses unique characteristics, such as motifs 3, 4, 6, 7, and 8 being exclusive to the CYC/TB1 branch, and motif 10 being present in all CIN members, highlighting the complementary roles these motifs may play in protein function.

In the analysis of conserved domains, we identified that *PbTCP* transcription factors possess a typical bHLH domain, with high conservation within subfamilies. A notable finding is the significant loss of amino acids in the loop and Helix 2 regions of the *PbTCP14* protein. Compared to *PbTCP14*, *PbTCP15* contains additional motifs 8 and 3, suggesting that the loss of these motifs in *PbTCP14* may influence plant growth, development, or stress responses. The second conserved region in CYC/TB1, the R domain, is known to be rich in polar residues such as arginine, lysine, and glutamic acid. The type and sequence of amino acids in this domain significantly affect its hydrophilicity [53], which is consistent with the high hydrophilicity observed in other *P. bournei* gene families [54,55,56,57]. This specificity may be related to the adaptation of *P. bournei* to its moist environment.

Gene duplication events play a crucial role in providing genetic material and promoting gene evolution. Our synteny analysis of the 29 *PbTCP* genes revealed that they all exhibit syntenic relationships with multiple genes. Among the 15 segmental duplication events, nine occurred in the PCF subfamily, four in the CIN subfamily, and only two in the CYC/TB1 subfamily. Previous studies have identified 12 segmental duplication events in ginger, and the number of *PbTCP* segmental duplications far exceeds that in *Orchard grass* [58], *Dendrobium officinale* [45], and *Capsicum annuum* [3], suggesting a potential evolutionary advantage in *P. bournei* that may enhance its survival and reproductive success. Additionally, interspecies synteny analysis between *P. bournei* and *A. thaliana*, *Z. mays*, and *P. trichocarpa* revealed lower synteny and sequence similarity with rice, potentially reflecting differences in evolutionary history, gene family expansion, and recombination.

To further explore the response of *PbTCP* genes to stress, we analyzed their expression profiles and cis-acting elements. *PbTCP* genes are expressed in roots, stems, and leaves, with the highest expression levels in the PCF subfamily. In contrast, most genes in the other two subfamilies are reduced, like the expression pattern observed in *Helianthus annuus* [12]. However, unlike *H. annuus*, where only a few PCF genes are highly expressed, *P. bournei* shows significantly higher expression in the PCF subfamily. In *Capsicum annuum*, the CIN subfamily exhibits the highest expression [3], indicating species-specific differences in *TCP* transcription factor expression. *PbTCP16* and *PbTCP23* show prominent expression in roots, stems, and leaves, with *PbTCP23* notably responsive to MeJA, which promotes defensive protein production in plants to concentrate resources against environmental stress [59], suggesting their significant roles in abiotic stress response. Additionally, *PbTCP18*, a member of the CIN subfamily, is upregulated in leaves and roots, like the expression patterns of *AtTCP2* and *AtTCP3*, which are associated with active cell division in *floral meristems* [53]. Given the strong phylogenetic relationship between *PbTCP18* and *AtTCP2*/*AtTCP3*, it is plausible that *PbTCP18* promotes meristematic tissue differentiation in leaves and roots.

**Figure 12:**
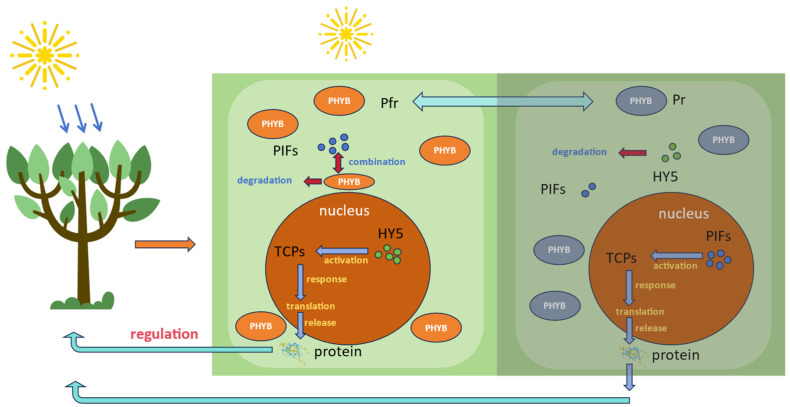
The different responses of PIFs and HY5 under Pfr and Pr form. Note: Light promotes the degradation of PIF and the expression of HY5 in the nucleus through photosensitive pigments (Pfr); Under dark conditions, the Pr form promotes the transcription and accumulation of PIFs and the degradation of HY5, which together regulate the normal physiological state of plants.

We conducted abiotic stress experiments on *PbTCP16*, *PbTCP23*, *PbTCP7*, *PbTCP29*, *PbTCP14*, and *PbTCP15*, representing the three subfamilies. The results revealed differential expression of these genes under high temperature, drought, light stress, and shading conditions. Genes involved in stress response predominantly belonged to the CYC/TB1 subfamily, differing from other species like *Betula platyphylla* and *Phyllostachys edulis* [60,61], where the PCF subfamily showed more active expression under stress. Notably, *PbTCP16* exhibited downregulation across all stress conditions, possibly due to its low confidence in secondary structure prediction, making it less favorable for stress response. *PbTCP14* and *PbTCP15*, on the other hand, demonstrated strong expression under various stresses, particularly in response to light and shading, where they showed significantly higher expression than other representative genes, indicating greater resistance to light and dark conditions. Previous studies have shown that *TCP* transcription factors activate PIFs under shade conditions, further promoting auxin synthesis and plant growth (Figure 12) [24,25]. The cis-element analysis also revealed that all *PbTCP* transcription factors are highly expressed in response to light stress. In *A. thaliana*, *TCP2* positively regulates key factors in the light signaling pathway, such as HY5 and HYH, altering plant morphology under light conditions [62]. Similar conclusions have been drawn in other studies [51], highlighting the critical role of *TCP* transcription factors in adapting to changes in environmental light conditions.

## 4. Materials and Methods

### 4.1. Identification of PbTCP Genes in P. bournei

The genome assembly file for *P. bournei* was downloaded from the NCBI Conserved Domain Database (NCBI-CDD) [63,64]. To search for *TCP* gene candidates within the *P. bournei* genome, we utilized a specific Hidden Markov Model (HMM) of the *TCP* transcription factor (Pfam number: PF03634) [64]. Subsequently, we utilized TB tools to compare the protein sequences of *A. thaliana* and *P. bournei* with default parameters. A total of 30 genes were identified, while 29 belong to the *TCP* transcription factor family, and were renamed according to their position on the chromosomes. Additionally, we used Batch CD-Search (default parameters) from the website (https://www.ncbi.nlm.nih.gov/Structure/bwrpsb/bwrpsb.cgi (accessed on 6 August 2024))) to confirm the conserved domain of *PbTCP* [65]. The physicochemical characteristics of all *PbTCP* proteins, encompassing amino acid number (size), molecular weight (MW), theoretical isoelectric point (pI), instability index, aliphatic index, and grand average of hydropathicity (GRAVY), were assessed through the utilization of the ExPASy online tool (https://www.expasy.org/ (accessed on 6 August 2024)). Subsequently, the website Cell-PLoc 2.0 (http://www.csbio.sjtu.edu.cn/bioinf/Cell-PLoc-2/ (accessed on 6 August 2024)) was employed to forecast the subcellular localization of the *PbTCP* proteins.

### 4.2. Phylogenetic Analysis

Sequence alignment was conducted on 29 amino acid sequences of the *PbTCP* protein using the MUSCLE function, with default parameters, in MEGA 7.0.21 [66]. The multiple sequence alignment results were then swiftly trimmed using Quick Run TrimAL in TBtools (V1.120) [67]. Subsequently, a dependable intraspecific phylogenetic tree was generated using the trimmed sequences. Furthermore, a phylogenetic tree of *A. thaliana* and *P. bournei* was constructed using the neighbor-joining approach (NJ) with 1000 bootstrap replications in MEGA 7.0.21. The interspecific phylogenetic tree was improved with the Evolview website (https://www.evolgenius.info/evolview/#/treeview (accessed on 6 August 2024)). Additionally, Jalview 2.11.2.7 was used to enhance the sequence alignment results [68].

### 4.3. Protein Motifs Analysis and Gene Structures, Conserved Domain

Using the Multiple Em for Motif Elicitation (MEME) suite (http://meme-suite.org/tools/meme (accessed on 6 August 2024)) [69], conservative motifs of the protein sequences were exhibited with two parameters: the maximum motif number was 10 and the motif site occurrences were distributed at zero or one per sequence. Distribution data for exons and introns was obtained from the genome GFF files of *P. bournei*. The conserved domain of *PbTCP* proteins was uploaded and verified by the NCBI-CDD database. Finally, the intron–exon structure, conserved domain, and 10 motifs of the *PbTCP* proteins were visualized using TBtools (V1.120) [67].

### 4.4. Chromosomal Location, Gene Duplication, and Collinearity Relationship

The location of the *PbTCP* genes was determined using the GFF annotation files of the *P. bournei* genome [63]. Utilizing MG2C v2.1 (http://mg2c.iask.in/mg2c_v2.1/ (accessed on 7 August 2024)), we generated a pattern of chromosome location. Gene duplication models of the *PbTCP* family gene were identified and analyzed using Tbtools. To obtain the genome files of four other plant species, namely Arabidopsis, rice, and poplar, we retrieved them from the NCBI. The collinearity relationships between *PbTCP* and the three mentioned species were analyzed and visualized using the “Advanced Circos” tool in Tbtools.

### 4.5. Cis-Elements in the Promoter and Expression Analysis of PbTCP Genes

The 2000 bp upstream sequence of *PbTCPs* was extracted and served as the promoter sequence used to identify cis-elements and prediction using PlantCARE (https://bioinformatics.psb.ugent.be/webtools/plantcare/html/ (accessed on 7 August 2024)) [70]. The positions and numbers of the identified cis-elements were visualized by Tbtools. RNA-seq data of different tissues in *P. bournei* were downloaded from the NCBI database using BioProject accession number PRJNA628065 [65].

### 4.6. Plant Materials and Abiotic Stresses Treatment

Seedlings were carefully selected from one-year-old *P. bournei* specimens. Before planting the seedlings, the soil was prepared by mixing peat moss, humus soil, sandy soil, and perlite in the ratio of 5:2:2:1, and the organic matter content was between 2.57% and 6.07%. The annual average temperature in the growth area was between 16 °C and 20 °C, the annual precipitation was between 900 mm and 2100 mm, and the annual relative humidity was about 77%. In our experiment, samples were taken from the drought and heat treatment groups at 0, 4, 8, 12, and 24 h, from the light treatment group at 0, 24, 48 and 72 h, and from the dark treatment group at 0, 12, 24, 48 and 72 h. The seedlings collected at 0 h were used as the control group. Each treatment has three biological replicates, meaning each treatment includes three individuals (at 0 h). A sample taken from one seedling (3–5 mature leaves) constitutes one biological replicate. Each group was subjected to the appropriate experimental conditions. During the stress treatment process, the parameters set in the artificial climate chamber were as follows: the light period was 12 h/d, LED lamps were used for lighting, the photosynthetically active radiation was set at 1200 μmol·mol^−1^·s^−1^, and the temperature was 25 °C. The experimental plan was designed to simulate drought stress, in which the treatment groups were transplanted into beakers containing 10% PEG 6000, which was a means of simulating drought conditions. For temperature treatment, individuals were incubated at 40 °C. For light stress, the control group was sampled under natural conditions at 0, 24, 48, and 72 h, while the treatment group was continuously exposed to light and sampled at 0, 24, 48, and 72 h. For dark treatment, except for the control group, samples were collected at 12, 24, 48, and 72 h in darkness. After the treatment was completed, the leaves were immediately collected and stored at −80 °C in liquid nitrogen for subsequent RNA extraction.

### 4.7. RNA Extraction and qRT-PCR Analysis

RNA was extracted from both control and stress-treated leaf tissue samples using a HiPure Plant RNA Mini Kit (Magen), followed by cDNA synthesis with a PrimeScript RT reagent Kit (Perfect Real Time) (TaKaRa). qRT-PCR was then conducted to determine gene (PCF: *PbTCP16* and *PbTCP23*, CIN: *PbTCP7* and *PbTCP29*, CYC/TB1: *PbTCP14* and *PbTCP15*) expression profiles in response to stress. The qRT-PCR experiment utilized specific primers which were designed through the Primer 3 website (http://bioinfo.ut.ee/primer3-0.4.0/ (accessed on 7 August 2024)). PbEF1α was selected as the reference gene (GenBank number, KX682032.1) [43]. The raw Cq values were assessed using the 2^−ΔΔCT^ method and then compared to the reference gene [71]. All experiments were conducted with three biological replicates (Taking one sample (three to five mature leaves) from each of three seedlings) and three technical replicates. The relative levels of gene expression were analyzed using one-way ANOVA, with multiple comparisons with the control group at a significance threshold of 5%. The expression graphs were generated via GraphPad Prism 8.3.0 software.

## 5. Conclusions

This study identified 29 *PbTCP* genes in *P. bournei* and analyzed their properties, relationships, structures, functions, and expression patterns. The *TCP* gene family was classified into two classes: Class I (PCF) and Class II (CIN and CYC/TB1). The PCF subgroup was the most conserved, while the CYC/TB1 subgroup exhibited the greatest evolutionary divergence. PCF genes showed notable expression in roots, stems, and leaves, suggesting a potential involvement in growth and development. Further analysis revealed that *PbTCP14* and *PbTCP15* had high expression in both light and dark environments, suggesting a potential role in light stress resistance. However, the mechanisms by which *TCP* transcription factors regulate growth and development in *P. bournei* require further investigation. This study systematically analyzed the expression patterns of *TCP* genes in different tissues and their response to abiotic stress, providing valuable insights into their role in regulating plant growth and development. These findings lay the foundation for future research on stress resistance and functional genomics in this species.

## Figures and Tables

**Table 1:** Analysis of amino acid sequence characteristics of *TCP* gene family of *Phoebe bournei* (Hemsl.).

Gene Accession	Protein Name	AA/aa	MW/kDa	pI	II	AI	GRAVY	Subcellular Localization
OF00845-RA	PbTCP1	443	46.01	6.67	59.42	66.5	−0.491	Nucleus
OF19791-RA	PbTCP2	301	33.72	8.27	64.22	71.3	−0.642	Nucleus
OF22744-RA	PbTCP3	259	28.65	9.88	61.33	58.84	−0.777	Nucleus
OF11465-RA	PbTCP4	295	31.83	6.72	51.46	63.53	−0.693	Nucleus
OF11860-RA	PbTCP5	322	36.06	9.27	55.39	51.61	−0.774	Nucleus
OF04159-RA	PbTCP6	583	63.36	5.73	54.96	73.95	−0.54	Nucleus
OF04032-RA	PbTCP7	410	45.38	8.63	51.76	69.46	−0.74	Nucleus
OF25895-RA	PbTCP8	364	39.11	8.79	61.59	67.86	−0.563	Chloroplast, Nucleus
OF25557-RA	PbTCP9	679	72.15	9.49	37.11	92.99	−0.092	Mitochondrion
OF23858-RA	PbTCP10	177	17.80	10.15	69	66.89	−0.117	Nucleus
OF23646-RA	PbTCP11	277	30.78	7.63	44.67	74.69	−0.307	Nucleus
OF23645-RA	PbTCP12	377	42.23	9.51	43.12	71.17	−0.62	Nucleus
OF23643-RA	PbTCP13	435	48.75	8.94	42.58	78.23	−0.575	Nucleus
OF23640-RA	PbTCP14	231	25.55	9.65	45.24	58.74	−0.943	Nucleus
OF23639-RA	PbTCP15	438	48.41	9.57	48.37	69.06	−0.654	Nucleus
OF24987-RA	PbTCP16	250	25.86	6.11	48.73	71.8	−0.399	Nucleus
OF21041-RA	PbTCP17	440	45.75	7.31	63.06	62.25	−0.453	Nucleus
OF01693-RA	PbTCP18	438	47.80	6.58	47.43	62.95	−0.743	Nucleus
OF02672-RA	PbTCP19	331	37.11	9.13	42.31	61.66	−0.872	Nucleus
OF11303-RA	PbTCP20	406	44.71	9.48	51.89	70.86	−0.596	Nucleus
OF02247-RA	PbTCP21	363	38.35	8.11	58.39	65.21	−0.572	Nucleus
OF05103-RA	PbTCP22	464	51.05	5.95	57.67	57.87	−0.809	Nucleus
OF18329-RA	PbTCP23	238	25.28	8.89	51.85	76.34	−0.321	Nucleus
OF26947-RA	PbTCP24	309	32.91	5.68	53.61	73.88	−0.469	Nucleus
OF00332-RA	PbTCP25	290	30.59	9.39	66.12	72.07	−0.364	Nucleus
OF29850-RA	PbTCP26	344	36.00	9.55	68.42	72.12	−0.426	Nucleus
OF21638-RA	PbTCP27	378	40.60	6.64	56.62	65.61	−0.536	Nucleus
OF17792-RA	PbTCP28	234	24.43	9.98	56.51	78.08	−0.279	Nucleus
OF09077-RA	PbTCP29	414	45.10	6.57	69.1	57.39	−0.746	Nucleus

Note: AA: number of amino acids; MW: molecular weight; pI: theoretical isoelectric point; II: instability index; AI: aliphatic index; GRAVY: grand average of hydropathicity.

## Data Availability

Data are contained within the article.
